# A comprehensive operative risk assessment driving the application of major and emergency surgery in octogenarians

**DOI:** 10.14814/phy2.70214

**Published:** 2025-02-18

**Authors:** Francesco Puccetti, Alessandro Francesco Armienti, Stefano Turi, Lorenzo Cinelli, Riccardo Rosati, Ugo Elmore, OSR CCeR Collaborative Group, Lavinia Alessandra Barbieri, Silvia Battaglia, Andrea Cossu, Lorenzo Gozzini, Davide Socci, Elio Treppiedi, Alessia Vallorani

**Affiliations:** ^1^ Department of Gastrointestinal Surgery IRCCS San Raffaele Scientific Institute Milan Italy; ^2^ School of Medicine Vita‐Salute San Raffaele University Milan Italy; ^3^ Department of Anesthesiology and Intensive Care IRCCS San Raffaele Scientific Institute Milan Italy

**Keywords:** cancer surgery, emergency, frailty, functional reserve, operative risk

## Abstract

Medical decisions driving the clinical management of octogenarians who require either major or urgent surgery still depend on the patient's age rather than individual functions. This report created the privileged opportunity to illustrate the clinical effectiveness of a comprehensive function‐based assessment. This was the case of an 83‐year‐old gentleman presenting with severe malnutrition and debility due to esophageal cancer. Multidimensional assessments were systematically performed to design the best‐tailored therapeutic strategy, including prehabilitation, elective esophagectomy, and emergency laparotomy with ileocolic resection for postoperative hemorrhagic shock due to an occult colonic tumor. This clinical case highlights the need for a systematic and comprehensive assessment of fragile octogenarians, allowing accurate patient evaluation, identification of areas of functional optimization, and establishment of the most appropriate therapeutic decisions.

## 1 INTRODUCTION

Given the considerable rise in global life expectancy, patients requiring in‐hospital invasive treatments have been gradually aging (Fowler et al., 2019). The increasing proportions of elderly patients within the surgical population has moved healthcare providers and multidisciplinary teams to early identify more specific criteria for operative risk assessment. Accordingly, the patient's age at the moment of surgery turned out to be a non‐univocal indicator of fitness to surgery, while the balance between patient's biological characteristics and type of surgical procedures may provide elements for a more precise operative risk assessment. Frailty is defined as the increased vulnerability resulting from reductions in physiological reserve and multisystem functions and represents the main determinant of the operative stress response in the elderly (Lin et al., 2016). A prompt and comprehensive multidimensional assessment (MDA) is the key element for allocation of resources, identification of optimization areas, and careful choices in geriatric or palliative settings. This report describes the clinical case of an octogenarian who, further to the precise definition and enhancement of his functional reserve, underwent tailored approaches of esophagectomy for cancer and emergency laparotomy afterward.

## 2 CASE PRESENTATION

**FIGURE 1:**
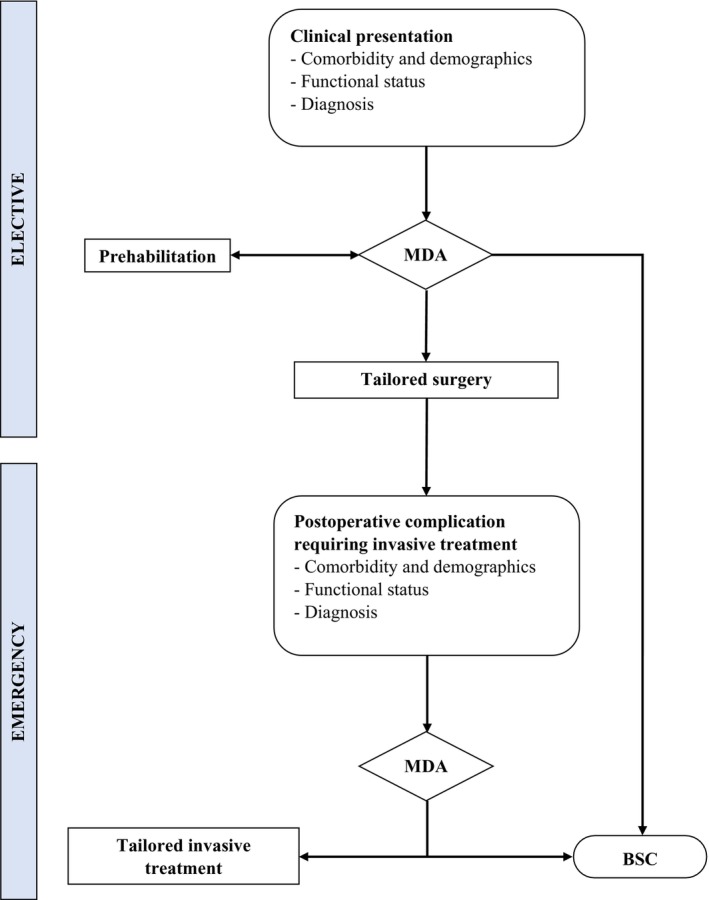
Frailty–based multidimensional decisional approach. BSC, best supportive care; MDA, multidimensional assessment.

The present case report has been reported in compliance with the Consensus‐based Clinical Case Reporting Guidelines (CARE). An 83‐year‐old gentleman presented with significant medical history (i.e., severe chronic obstructive pulmonary disease, insulin‐dependent diabetes, and hypertension) and physical deterioration due to malnourishment (i.e., complete dysphagia with about 8 kg weight loss in 9 weeks) secondary to a locally advanced esophageal adenocarcinoma. According to our dedicated institutional board a MDA was performed, involving different medical specialists, such as surgeon, anesthetist, oncologist, physiotherapist and nutritionist. Regarding the several patient's comorbidities, age and disease‐related disablement, a dedicated thorough respiratory, cardiac and performance status assessment was performed through specific tests (i.e. Karnofsky performance scale, spirometry, 6‐min walk test, nutritional risk screening score, Duke Activity Status Index) (Inoue et al., 2020; Wijeysundera et al., 2018, 2020) in order to define the actual patient's functional impairment guiding the medical decision (Table 1). The initial MDA (Figure 1), suggested an 8‐week prehabilitation program, including muscular and respiratory physical exercises with combined artificial (i.e., enteral + parenteral) nutrition (Table 1). Prehabilitation was mostly conducted at home with relatives' involvement and under the physiotherapist's guidance. The intermediate MDA registered general improvements in nutritional status (4 kg weight gain) and respiratory capacity (Table 1). Therefore, the patient was submitted to esophageal resection with a minimized surgical approach (i.e., transhiatal) to avoid chest opening and single‐lung ventilation. Transhiatal esophagectomy was performed with abdominal and lower‐mediastinal lymphadenectomy, cervical end‐to‐end esophagogastric anastomosis, feeding jejunostomy, and large right groin hernia repair. Operation was uneventful (307 min; blood loss of 210 mL), and ICU monitoring was not necessary after surgery. Postoperative recovery was complicated by a conservatively treated anastomotic leak and bilateral pleural effusion with pneumonia, requiring antibiotics and non‐invasive oxygen support. However, the most significant complication was a hemorrhagic shock caused by acute colonic bleeding originating from an occult cecum adenocarcinoma. To drive the urgent treatment strategy an additional MDA was performed and, given the functional reserve reduction, the patient was submitted to palliative ileocecal resection with minimization of both surgical approach (resection restricted to the source of bleeding, without radical lymphadenectomy) and anesthesia management (exclusive awake regional anesthesia) (Table 1). Emergency laparotomy was uneventful (119 min; blood loss of 120 mL), and ICU monitoring was not necessary after surgery. Afterward, postoperative recovery did not present further complications, and the patient was discharged at home after pneumonia resolution. After 90 days, the patient was alive and returned to normal daily activities with regular oral feeding along with 500 kcal nutritional supplementation, daily administered through the feeding jejunostomy.

**TABLE 1:** Risk assessment in elderly/frail patients.

	At the diagnosis	Elective major surgery	Urgent surgery
Age	83	83	83
ASA physical status classification	3	3	3
NRS	5	3	3
Karnofsky performance scale	50%	70%	30%
Respiratory function impairment^a^	Moderate	Mild^d^	Severe^e^
Cardiac function impairment^b^	Mild	Mild	Mild
Functional impairment^c^	Severe	Mild^d^	Severe^e^
Decision	Non‐fit for surgery without an adequate prehabilitation	Fit for minimized surgery (transhiatal esophagectomy with two‐lung ventilation) with possible ICU recovery for postoperative monitoring	Fit for minimized surgery (no lymphadenectomy) and minimized anesthesia (regional analgesia), and possible ICU recovery for postoperative monitoring

Abbreviations: ASA, American Society of Anesthesiology; DASI, questionnaire; NRS, Nutritional Risk Screening.

^a^ Based on respiratory symptoms, comorbidity and spirometry.

^b^ Based on Revised cardiac risk index (Lee Criteria), cardiologist evaluation and NYHA class.

^c^ Based on 6‐min walk test and strength and coordination evaluation, DASI questionnaire.

^d^ Symptom resolution and improvement of respiratory assessment, improvement of functional capacity after prehabilitation.

^e^ Due to the combination of acute and chronic deficit worsening because of postoperative complications.

## 3 DISCUSSION

**FIGURE 2:**
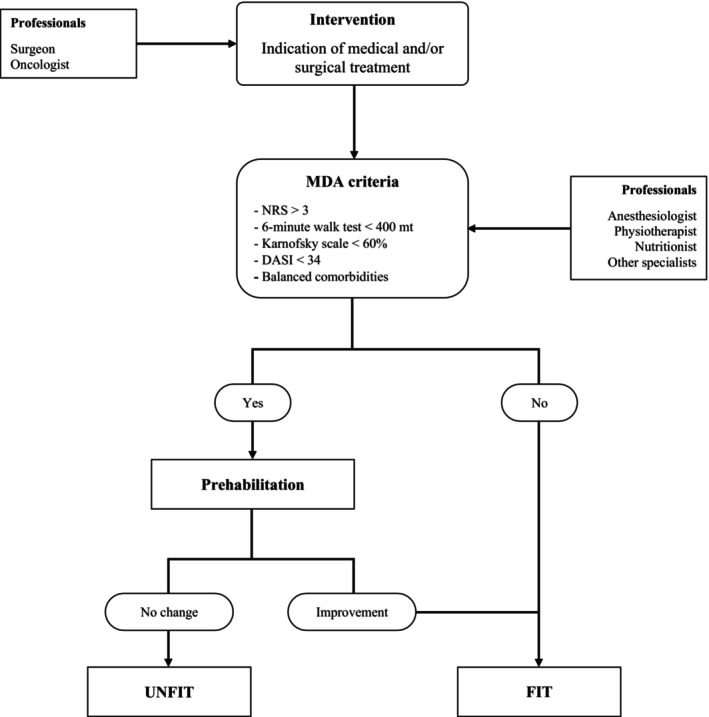
Stepwise procedure of Multidimensional Assessment (MDA).

Increasing evidence has been supporting the application of major surgery to elderly population, including the most impactful operations, such as gastroesophageal resections (Laurent et al., 2022). It has been achieved that age does not represent an exclusive criterion limiting indications to surgery, while the choice should be made with regard to objective parameters, the need for surgery, and possible alternatives. The main role of the MDA (Figure 2) is to highlight the most relevant biological determinants in order to identify possible areas of optimization and to establish individual treatments tailored to patient functions. In patients undergoing major abdominal surgery, enhanced recovery after surgery (ERAS) guidelines recommend a careful preoperative cardiovascular and respiratory evaluation, to optimize the management of existing comorbidities and to plan the best intraoperative conduct and postoperative monitoring (Feldheiser et al., 2016). The development of multidisciplinary pathways to assess and improve patients' medical conditions should be especially encouraged in frail patients (Pang et al., 2021). Despite the low level of evidence, multimodal prehabilitation programs for patients receiving esophageal cancer resections appear to be associated with improvements in functional capacity (Minnella et al., 2018). In a recent prospective study by Halliday et al. (2021), esophageal cancer patients with a low baseline fitness were more likely to increase their functional capacity after prehabilitation, although these patients also presented a lower level of adherence to exercise programs. In our experience, effective prehabilitation programs can be successfully achieved through a close clinical monitoring only. The MDA implementation can be profitably extended to acute conditions and emergency surgery, demonstrating that operative stress minimization (including both surgical and anesthesiology components) aligns with treatment optimization and tailoring. We extensively explained the operative stress physiology and recovery after emergency treatments, showing management limitations or optimizations (Puccetti et al., 2023). In a recent meta‐analysis, Hajibandeh et al. (2021) reported the significantly high operative risk of octogenarians undergoing emergency general surgery, identifying risk characteristics correlating with mortality. As commented by Rubin et al. (2021), the type of anesthesia also plays a relevant role and contributes to the operative stress composition. Both general and neuraxial awake anesthesia present respectively advantages and contraindications in these specific groups of patients. Considering the severe impairment in respiratory function (i.e., recent pneumonia with the need for oxygen support), we decided to manage the emergency procedure only through a loco‐regional anesthesia, placing a thoracic epidural catheter. Also, a goal‐directed fluid management allowed the intraoperative maintenance of hemodynamic stability, although an adequate level of patient collaboration has to be ensured before treatment.

The main limitation of this case report is the single‐patient analysis, which includes multiple elements of clinical peculiarities and potentially reduces the generalizability of these results. However, clinical aspects of perioperative management, functional assessment, and accurate patient‐tailored diagnostics could be effectively shared by other surgical units, who wondered about the best way to treat any delicate category of patients.

The present brief report suggests that octogenarians represent a frail category of patients, carrying a high risk for postoperative morbidity and early mortality. Palliative oncological surgery might include either major or urgent operations, as long as this decision is carefully considered and endorsed by a thorough multidimensional assessment. Accordingly, the choice for invasive approach demands the absence of possible therapeutic alternatives, while every potential consequence must be accurately and timely pondered within a comprehensive counseling to patients and relatives. In conclusion, either emergency or elective major surgery in elderly patients should not be excluded a priori, but these can be tailored/minimized according to the patient functional resources at a specific time. ERAS protocols and prehabilitation are strongly recommended in elderly since a high functional improvement can be attained in order to include the patient in invasive treatments. Therefore, specific and dedicated multidimensional assessment and medical protocols should be developed for elderly and frail patients.

## FUNDING INFORMATION

No funding information provided.

## CONFLICT OF INTEREST STATEMENT

No competing interests declared.

## ETHICS STATEMENT

The study was conducted in accordance with the Declaration of Helsinki, and approved by the Institutional Review Board of IRCCS San Raffaele Scientific Institute (Approval ID: 91/INT/2021, 30 June 2021).

## Data Availability

Data are available upon request.
